# Maternal morbidity and mortality from severe sepsis: a national cohort study

**DOI:** 10.1136/bmjopen-2016-012323

**Published:** 2016-08-23

**Authors:** Colleen D Acosta, David A Harrison, Kathy Rowan, D Nuala Lucas, Jennifer J Kurinczuk, Marian Knight

**Affiliations:** 1 National Perinatal Epidemiology Unit (NPEU), Nuffield Department of Population Health, University of Oxford, Oxford, UK; 2 Intensive Care National Audit & Research Centre (ICNARC), London, UK; 3 Department of Anaesthesia, Northwick Park Hospital, Harrow, Middlesex, UK

**Keywords:** sepsis, pregnancy, critical care, cohort study

## Abstract

**Objectives:**

To describe the incidence, characteristics and risk factors for critical care admission with severe maternal sepsis in the UK.

**Design:**

National cohort study.

**Setting:**

198 critical care units in the UK.

**Participants:**

646 pregnant and recently pregnant women who had severe sepsis within the first 24 hours of admission in 2008–2010.

**Primary and secondary outcome measures:**

Septic shock, mortality.

**Results:**

Of all maternal critical care admissions, 14.4% (n=646) had severe sepsis; 10.6% (n=474) had septic shock. The absolute risk of maternal critical care admission with severe sepsis was 4.1/10 000 maternities. Pneumonia/respiratory infection (irrespective of the H1N1 pandemic influenza strain) and genital tract infection were the most common sources of sepsis (40% and 24%, respectively). We identified a significant gradient in the risk of severe maternal sepsis associated with increasing deprivation (RR=6.5; 95% CI 4.9 to 8.5 most deprived compared with most affluent women). The absolute risk of mortality was 1.8/100 000 maternities. The most common source of infection among women who died was pneumonia/respiratory infection (41%). Known risk factors for morbidity supported by this study were: younger age, multiple gestation birth and caesarean section. Significant risk factors for mortality in unadjusted analysis were: age ≥35 years (unadjusted OR (uOR)=3.5; 95% CI 1.1 to 10.6), ≥3 organ system dysfunctions (uOR=12.7; 95% CI 2.9 to 55.1), respiratory dysfunction (uOR=6.5; 95% CI1.9 to 21.6), renal dysfunction (uOR=5.6; 95% CI 2.3 to 13.4) and haematological dysfunction (uOR=6.5; 95% CI 2.9 to 14.6).

**Conclusions:**

This study suggests a need to improve timely recognition of severe respiratory tract and genital tract infection in the obstetric population. The social gradient associated with the risk of severe sepsis morbidity and mortality raises important questions regarding maternal health service provision and usage.

**Strengths and limitations of this study**

- The study used comprehensive and nationally representative data with a large sample size and hence results have high external validity.
- This study had a robust case definition and results were validated in sensitivity analyses using obstetric-specific systemic inflammatory response syndrome criteria.
- The data used were from 2008 to 2010, which may make results less generalisable to current practice.
- Comparison of cohort data to aggregated national data, and low statistical power due to the small number of deaths, precluded multivariable risk modelling for the outcomes of morbidity and mortality.
- Data on the characteristics of non-reporting hospitals were not available; thus, it was not possible to conduct a sensitivity analysis to assess differences between reporting and non-reporting hospitals.

## Introduction

Even in an era of modern antibiotics and advanced medical care, the incidence of sepsis has increased dramatically worldwide.^1^ ^2^ Sepsis is a leading cause of direct maternal death in the UK,^3^ ^4^ and is a major cause of direct maternal death in other high-resource and low-resource countries.^5–8^ While the absolute risk of death from maternal sepsis is low in the UK (2.0/100 000 maternities^4^), a study using the UK Obstetric Surveillance System (UKOSS), which captures data from obstetrician-led maternity units, found that the magnitude of severe morbidity is ∼50 times greater.^9^ The rate of maternal critical care admissions with severe sepsis in the UK, including women admitted from maternity units as well as other services among women outside the immediate period of labour and delivery, is however unknown.

In countries that have relatively low maternal mortality rates, identification of risk factors for severe morbidity is critical to target points of intervention before progression to more serious outcomes. The UKOSS study investigated risk factors for severe morbidity; however, the study cohort was restricted to maternity units due to the nature of the data collection system.^9^ Studies of maternal sepsis are also made more challenging due to the normal physiological changes of pregnancy, which overlap with some of the pathophysiological changes of sepsis. Currently, there has been no national level study of the incidence or risk factors for admission to critical care with severe maternal sepsis in the UK.

The objectives of this study were to describe the incidence and characteristics of pregnant or recently pregnant women who had severe sepsis within the first 24 hours following critical care admission in the UK, and to evaluate the risk factors for severe sepsis morbidity on a national population level, in order to inform strategies to improve prevention and outcomes.

## Methods

### Data sources

This study was conducted using data on women admitted to critical care units from the Intensive Care National Audit & Research Centre (ICNARC) Case Mix Programme (CMP) database. The CMP is the national clinical audit for adult critical care units (including intensive care and combined intensive care and high dependency units) in England, Wales and Northern Ireland, and is coordinated by ICNARC. The CMP database contains pooled case mix data, collected from the first 24 hours following admission to the critical care unit, and outcome data on consecutive admissions to units participating in the CMP.^10^ The CMP database has been independently assessed to be of high quality and regular assessment of data quality is ongoing.^11^ Data validation procedures and variable definitions have been described in previous studies.^10^ ^12^ All data used in this study were validated and from units that had been reporting to the CMP for at least 6 months. Support for the collection and use of patient-identifiable data without consent was obtained under section 251 of the National Health Service (NHS) Act 2006 (approval number PIAG 2–10(f)/2005).

National statistics were used for comparison with CMP data in this study. Data on maternal age, deprivation (measured using the Index of Multiple Deprivation (IMD)), multiple births and stillbirths were obtained from the Office for National Statistics (ONS) for England and Wales,^13^ and the Northern Ireland Statistics and Research Agency (NISRA).^14^ Data on ethnic group were obtained from ONS and extrapolated for Northern Ireland based on the reported ethnic population distribution in the region.^15^ Data on mode of delivery were obtained from Hospital Episode Statistics,^16^ StatsWales^17^ and extrapolated for Northern Ireland based on published rates of mode of delivery in the region.^18^

Scottish critical care units do not participate in the CMP and therefore data from Scotland are not included in the study. Scotland accounts for ∼6% of all UK maternal critical care admissions, 2% of maternal sepsis critical care admissions^19^ and 7.3% of maternities.^20^ Data comprising this study, therefore, were considered representative of the UK maternity population, and results are generalised to that of the UK in discussion.

### Study design and case definition

This was an anonymised cohort study of all pregnant and ‘recently pregnant’ women who were reported in the CMP from 2008 to 2010, and who were either admitted with or developed severe sepsis within the first 24 hours of admission. Pregnant women were still pregnant on admission to critical care. Recently pregnant women were defined as having been pregnant within 42 days prior to admission to the critical care unit regardless of how the pregnancy ended (live birth, stillbirth, miscarriage or termination). Mortality was measured by death at ultimate discharge from acute hospital, irrespective of direct or indirect causes. Severe sepsis was defined according to a modified version of the protein C worldwide evaluation in severe sepsis (PROWESS) clinical trial definition:^21^ diagnosis of infection as primary or secondary reason for critical care unit admission and at least three systemic inflammatory response syndrome (SIRS) criteria and evidence of at least one organ system dysfunction. Septic shock was defined as severe sepsis with cardiovascular organ system dysfunction^22^ (systolic blood pressure <90 mm Hg or mean arterial pressure <70 mm Hg). Readmissions of women to critical care during the same hospital stay were excluded to avoid double counting.

### Sample size and statistical analyses

The sample size of this study is governed by the rate of critical care unit reporting coverage during the study period. In 2008, 2009 and 2010, coverage of the database was 65.0%, 75.2% and 80.2% of critical care units in England, Wales and Northern Ireland reported to the CMP, respectively, with a total of 198 critical care units contributing data during this time. The total number of maternal critical care admissions with severe sepsis was estimated by extrapolating from the number of severe maternal sepsis admissions observed in the CMP database each year, based on the CMP reporting coverage rate for each year and the total number of adult general critical care units in England, Wales and Northern Ireland. The absolute risk or incidence of maternal critical care admissions with severe sepsis and maternal critical care deaths from severe sepsis were estimated by dividing these extrapolated numbers by the total number of maternities obtained from ONS^13^ and NISRA.^14^ Imputation methods were not used to estimate the counts of severe maternal sepsis admissions and deaths from non-reporting hospitals because characteristics of these hospitals were unavailable and may have differed from reporting hospitals (ie, data may not have been missing at random).

Using observed CMP data (not extrapolated data), frequencies of demographic, clinical and delivery characteristics were tabulated for all cases. Rates were compared with nationally available data where possible. Using all maternities in England, Wales and Northern Ireland as the comparison group, the relative risk (RR) with 95% CI for maternal critical care admission with severe sepsis was calculated for each variable. Characteristics of cohort survivors were compared with those of non-survivors in a univariable logistic regression model.

A sensitivity analysis was finally performed using the obstetric-specific SIRS criteria defined by Waterstone *et al*.^23^ This was carried out in order to assess whether the more rigorous criteria were significantly associated with greater severity of sepsis or a different pattern of maternal characteristics. Using the Waterstone criteria, elevated heart rate is defined as >100 bpm (compared to >90) and an elevated white cell count is defined as >17 000 per µL (compared to >1200).

Stata statistical software V.11 (StataCorp, College Station, Texas, USA) was used for all analyses.

## Results

### Incidence

**Figure 1:**
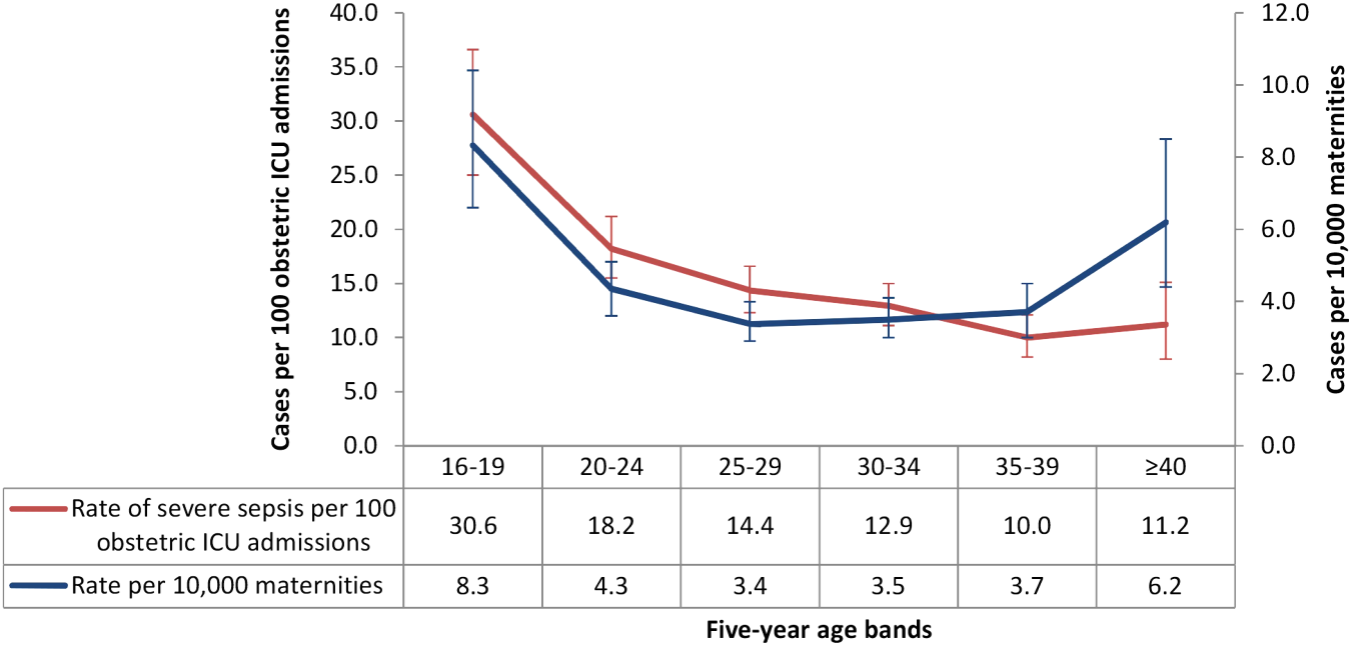
Rates and 95% CIs of severe sepsis among maternal critical care admissions and maternities by 5-year age bands from 2008 to 2010. ICU, intensive care unit.

In the CMP database between 2008 and 2010, there were 646 pregnant or recently pregnant women who met the case definition for severe sepsis, which represented 14.4% of maternal critical care unit admissions; 10.6% (n=474) had septic shock. Extrapolating from these figures based on the database coverage, 873 women in England, Wales and Northern Ireland were estimated to have had severe sepsis requiring critical care over the 3-year period, and 642 were estimated to have had septic shock. The estimated absolute risk of maternal critical care admission with severe sepsis was 4.1/10 000 maternities (95% CI 2.9 to 5.6). Sepsis admission rates were the highest among women aged 16–19 years (figure 1). One in three pregnant or recently pregnant women in this age category admitted to critical care had severe sepsis.

### Risk factors for admission to critical care with severe maternal sepsis

**Figure 2:**
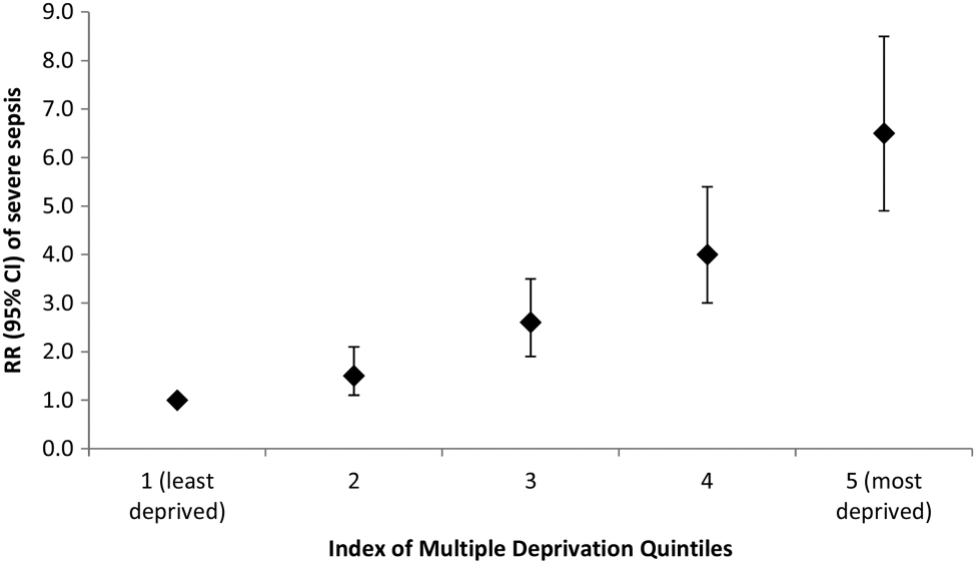
Relative risks and 95% CIs of severe sepsis according to deprivation quintiles (reference group=1st IMD quintile). IMD, Index of Multiple Deprivation; RR, relative risk.

Characteristics of the cohort are listed in table 1. Women had a significantly increased risk of admission to critical care with severe sepsis if they were aged <20 or ≥40 years compared with women aged 25–29 years. Increased risk of severe sepsis was also significantly and progressively associated with lower socioeconomic status (figure 2). Of women who delivered by caesarean section (n=242), 33.1% (n=80) were admitted directly from theatre for an emergency indication; 1.7% (n=4) were admitted after elective surgery. The RR of admission to a critical care unit with severe sepsis for women who had a caesarean section compared with a vaginal delivery was 6.2 (95% CI 4.9 to 7.8).

**Table 1:** Characteristics and relative risks of severe sepsis among pregnant and recently pregnant women

Total	Critical care admissions	All maternities	Relative risk (95% CI)
	646 (100.0)	2 186 818	
Recently pregnant	413 (63.9)		
Maternal age, years (mean; SD)	28.3 (6.9)	29.0 (–)	
<20	80 (12.4)	129 167 (5.9)	**2.5 (1.9 to 3.3)**
20–24	133 (20.6)	419 944 (19.2)	1.3 (1.0 to 1.6)
25–29	148 (23.0)	602 716 (27.6)	1
30–34	153 (23.7)	600 694 (27.5)	1.0 (0.8 to 1.3)
35–39	95 (14.7)	352 364 (16.1)	1.1 (0.9 to 1.4)
≥40	36 (5.6)	81 933 (3.7)	**1.8 (1.2 to 2.6)**
Non-white ethnicity	154 (23.8)	609 250 (27.9)	0.9 (0.8 to 1.0)
Index of multiple deprivation (quintiles)*			
1 (least deprived)	63 (10.1)	589 784 (27.6)	1
2	75 (12.0)	475 573 (22.2)	**1.5 (1.1 to 2.1)**
3	109 (17.5)	395 137 (18.5)	**2.6 (1.9 to 3.5)**
4	148 (23.8)	346 002 (16.2)	**4.0 (3.0 to 5.4)**
5 (most deprived)	228 (36.6)	330 916 (15.5)	**6.5 (4.9 to 8.5)**
BMI, kg/m^2^ (median; IQR)* (N=312)	26 (22–30)		
History of immunosuppression	15 (2.3)		
Weeks gestation			
Antenatal (median; IQR)	26 (20–31)		
Postnatal (median; IQR)	38 (31–41)		
*Recently pregnant women only*			
Parity*			
0	193 (48.4)		
1	96 (24.1)		
≥2	110 (27.6)		
Assisted conception*	24 (8.1)		
Mode of delivery†			
Spontaneous vaginal	98 (25.9)	1 334 242 (61.0)	1
Assisted vaginal	28 (7.4)	273 340 (12.5)	1.4 (0.9 to 2.1)
Caesarean section	242 (64.0)	535 999 (24.5)	**6.2 (4.9 to 7.8)**
Unknown	10 (2.7)		
All multiple births (live births and stillbirths)	28 (7.6)	34 663 (1.6)	**4.4 (3.1 to 6.3)**
Pregnancy outcomes			
Live births	321 (77.7)		
Stillbirths	47 (11.4)	11 697 (0.5%)	**21.3 (16.3 to 27.9)**
1st/2nd trimester loss	25 (6.1)		
Ectopic pregnancy	10 (2.4)		
Other‡	2 (0.5)		
Unknown	10 (2.4)		
Hysterectomy*	20 (5.4)		
Days since delivery (median; IQR)	3 (0–8)		

Bold typeface indicates statistically significant results. Figures are N (%) unless otherwise stated.

1=Reference Group.

*Of those reported.

†National rates are total deliveries.

‡Two women each had one live birth and one stillbirth from the most recent pregnancy.

BMI, body mass index.

In assessing whether the rate of caesarean section may have contributed to the social gradient associated with the risk of severe sepsis (since there was significant overlap between the rate of deprivation and caesarean section within the cohort), it was found that compared with affluent women (IMD quintiles 1–2), deprived women (IMD quintiles 4–5) had a 1.9 (95% CI 1.2 to 3.1) times higher odds of caesarean section; of all women in the cohort who had a caesarean section, 64.3% (n=151) were deprived compared with 19.2% (n=45) who were affluent.

### Sources of infection and severity of illness

Source of infection could be identified from the primary reason for admission to the critical care unit for 598 women (92.6%). Frequencies of the reported source of infection and severity of illness among women admitted with severe sepsis are shown in table 2. The most common source of infection was pneumonia/respiratory infection (n=257; 39.8%). Of these, only 27 were identified as laboratory confirmed cases of AH1N1 influenza; 2009 and 2010 were AH1N1 influenza pandemic years. There was no significant difference in severity of illness (Acute Physiology and Chronic Health Evaluation (APACHE) II score) between sources of infection; however, women with pneumonia or respiratory infection had a significantly longer critical care unit length of stay compared with other causes (median length of stay=4 days, IQR=2–9 days; p<0.001). There was a significantly different distribution of sources of infection between pregnant and recently pregnant women. A significantly greater proportion of pregnant women had pneumonia (p<0.001), urinary tract infection/pyelonephritis (p<0.001) or appendicitis (p=0.04). In contrast, a significantly greater proportion of recently pregnant women had a genital tract infection (p<0.001), an infection arising from surgical trauma (p=0.02) or septicaemia (p<0.05).

**Table 2:** Characteristics of pregnant and postpartum women on admission to intensive care units in the UK from 2008 to 2010

Total	646 (100.0)
Source of infection	
Pneumonia (chest infection)	257 (39.8)
Genital tract	157 (24.3)
UTI/pyelonephritis	59 (9.1)
Surgical trauma	24 (3.7)
Septicaemia	20 (3.1)
Appendicitis	19 (2.9)
Other infection	62 (9.6)
Unknown	48 (7.4)
Number of organ system dysfunctions	
1	222 (34.4)
2	234 (36.3)
≥3	189 (29.3)
Organ system dysfunctions*	
Cardiovascular	475 (73.5)
Respiratory	381 (59.0)
Metabolic acidosis	345 (53.4)
Renal	50 (7.7)
Haematological	65 (10.1)
ICNARC physiology score (median; IQR)	14 (10–20)
APACHE II score (median; IQR)	12 (10–16)
Days in ICU (median; IQR)	2.8 (1.3–5.7)
Deaths	29 (4.6)

Figures are N (%) unless otherwise stated.

*Organ system dysfunctions are not mutually exclusive.

ICNARC, Intensive Care National Audit & Research Centre; ICU, intensive care unit; UTI, urinary tract infection.

Of all severe maternal sepsis admissions, 4.6% (N=29) died. The estimated absolute risk of acute hospital mortality of women admitted was 1.8/100 000 maternities (95% CI 1.1 to 2.8). Pneumonia/respiratory infection was the most common source of sepsis among women who died (N=12; 41.4%). Demographic and clinical characteristics of survivors and non-survivors are presented in table 3. Women aged ≥35 years, with ≥3 organ system dysfunctions, and those with respiratory, renal and/or haematological dysfunction had significantly higher unadjusted odds of dying of severe sepsis.

**Table 3:** Characteristics of survivors and non-survivors of severe obstetric sepsis following critical care admission

	Severe sepsis survivors	Severe sepsis deaths	Unadjusted OR (95% CI)	Adjusted OR (95% CI)
	n=610	n=29		
Recently pregnant	387 (63.4)	22 (75.9)	1.8 (0.76 to 4.3)	1.1 (0.42 to 3.0)
Maternal age, years				
<25	234 (38.4)	5 (17.2)	1*	1*
25–34	254 (41.7)	15 (51.7)	2.8 (0.99 to 7.7)	2.2 (0.71 to 7.0)
≥35	121 (19.9)	9 (31.0)	3.5 (1.1 to 10.6)	3.3 (0.94 to 11.2)
Non-white ethnicity	147 (24.1)	7 (24.1)	0.98 (0.39 to 2.5)	0.59 (0.21 to 1.6)
Deprivation (IMD quintiles 4 and 5)	354 (58.1)	17 (58.6)	1.02 (0.48 to 2.2)	**2.6 (1.03 to 6.7)**
BMI, kg/m­^2^				
Unknown	317 (52.1)	13 (44.8)	0.78 (0.17 to 3.7)	1.2 (0.15 to 9.1)
<25	126 (20.7)	3 (10.3)	1*	1*
≥25<30	90 (14.8)	7 (24.1)	3.3 (0.8 to 13.3)	**5.2 (1.4 to 18.9)**
≥30	76 (12.5)	6 (20.7)	3.5 (0.9 to 14.6)	**6.3 (1.5 to 27.0)**
History of immunosuppression	13 (2.1)	2 (6.9)	3.3 (0.73 to 15.7)	
*Weeks gestation*				
Antenatal				
≥37	16 (7.0)	0 (0.0)	–	
25–36	104 (47.5)	3 (42.9)	1*	
<25	99 (45.2)	4 (57.1)	1.4 (0.31 to 6.4)	
Postnatal				
≥37	202 (54.9)	14 (66.7)	1*	
25–36	114 (31.0)	3 (14.3)	0.38 (0.11 to 1.3)	
<25	52 (14.1)	4 (19.1)	1.1 (0.35 to 3.5)	
*Recently pregnant women only*				
Parity				
0	183 (48.9)	9 (42.9)	1*	
1	88 (23.5)	7 (33.3)	1.6 (0.58 to 4.5)	
≥2	103 (27.5)	5 (23.8)	1.0 (0.32 to 3.0)	
Assisted conception	24 (8.7)	0 (0.0)	–	
Mode of delivery				
Spontaneous vaginal	89 (23.6)	8 (36.4)	1*	
Assisted vaginal	28 (7.4)	0 (0.0)	–	
Caesarean section	227 (60.2)	12 (54.6)	0.59 (0.23 to 1.5)	
Termination	23 (6.1)	2 (9.1)	0.97 (0.19 to 4.9)	
Ectopic	10 (2.7)	0 (0.0)	–	
All multiple births	24 (7.0)	3 (15.0)	2.3 (0.64 to 8.6)	
Stillbirth(s)	42 (21.9)	4 (33.3)	1.8 (0.5 to 6.2)	
Hysterectomy	16 (4.6)	3 (15.0)	3.6 (0.96 to 13.7)	
<24 hours since delivery	104 (27.6)	8 (36.4)	1.5 (0.61 to 3.7)	
Source of infection†				
Pneumonia (chest infection)	216 (35.4)	12 (41.4)	1.3 (0.60 to 2.7)	
Intrauterine infection	69 (11.3)	2 (6.9)	0.58 (0.13 to 2.5)	
Pelvic infection	47 (7.7)	0 (0.0)	–	
UTI/pyelonephritis	43 (7.1)	0 (0.0)	–	
Septicaemia‡	17 (2.8)	2 (6.9)	2.6 (0.57-11.7)	
Number of organ system dysfunctions				
1	221 (36.2)	2 (6.9)	1*	
2	224 (36.7)	8 (27.6)	3.9 (0.83 to 18.7)	
≥3	165 (27.1)	19 (65.5)	12.7 (2.9 to 55.1)	
Organ system dysfunction§				
Cardiovascular	444 (72.8)	24 (82.8)	1.8 (0.68 to 4.8)	
Respiratory	349 (57.2)	26 (89.7)	6.5 (1.9 to 21.6)	**8.1 (1.8 to 36.0)**
Metabolic acidosis	322 (52.8)	18 (62.1)	1.5 (0.68 to 3.1)	
Renal	39 (6.4)	8 (27.6)	5.6 (2.3 to 13.4)	2.9 (0.94 to 9.3)
Haematological	52 (8.5)	11 (37.9)	6.5 (2.9 to 14.6)	**5.7 (2.0 to 16.0)**

Bold typeface indicates statistically significant results. Figures are numbers (%) of women.‘–’ indicates OR estimate not possible due to zero incidence in either the case or control group.

*Reference group.

†Reasons for admission with a >5% frequency.

‡Admitted with or onset during critical care admission.

§Organ system dysfunctions not mutually exclusive.

BMI, body mass index; IMD, Index of Multiple Deprivation; UTI, urinary tract infection.

### Sensitivity analysis using obstetric-specific SIRS criteria

In a sensitivity analysis using the obstetric-specific SIRS criteria defined by Waterstone *et al*,^23^ 77 (11.9%) women did not meet the more rigorous criteria; however, there were no significant changes in the distribution of case characteristics or sources of infection.

## Discussion

Severe sepsis and septic shock morbidity are common among pregnant and recently pregnant women admitted to intensive care (1 in 7 and 1 in 9 obstetric intensive care unit admissions, respectively). The rate of maternal death from ‘all-cause’ maternal sepsis is substantially higher than that from genital tract sepsis alone (1.8/100 000 vs 0.5/100 000 maternities^3^ ^4^), and similar to the rate of maternal mortality from all infectious causes in the UK in 2009–2012 (2.0/100 000 maternities; 95% CI 1.6 to 2.6).^4^ We further identified several findings with clinical and healthcare policy implications: pneumonia/respiratory infection is a leading source of sepsis irrespective of epidemic influenza periods; and there are major significant disparities in socioeconomic status and the risk of severe sepsis.

A strength of this study is that it used comprehensive and nationally representative data; this allowed for the study of risk factors for severe maternal sepsis with data of higher quality and greater clinical detail than could be achieved using other approaches such as hospital discharge data. The study also used a robust case definition, and results were validated in sensitivity analyses using the obstetric-specific SIRS criteria. Owing to its large sample size, the external validity of this study is extremely high. However, there are several limitations in this study. We do not have data on organisms presumed or confirmed to be the causes of sepsis. Comparison of cohort data to aggregated national data, and low statistical power due to the small number of deaths, precluded multivariable risk modelling for the outcomes of morbidity and mortality. All cases of maternal sepsis occurring in high dependency units within obstetric settings will not necessarily be captured within the ICNARC data. It was also not possible to conduct a sensitivity analysis to assess the characteristics of non-reporting hospitals as these data were not available. Although we generalise the results in this study to the entire UK, we cannot rule out that rates and risk factors of severe sepsis may differ for Scotland. However, since Scotland accounts for a small proportion of total and sepsis-specific critical care admissions, it is unlikely that inclusion of data from Scotland would significantly affect the results.

Forty per cent of women with severe sepsis had pneumonia/respiratory infection as the source of sepsis. This finding supports that of the recent UK and Ireland Confidential Enquiries into Maternal Deaths and Morbidity 2009–2012, which found that 54% of all maternal sepsis deaths were caused by influenza (N=36; 43%) or pneumococcal disease (N=9; 11%).^4^ In the present study, women with pneumonia/respiratory infection had a significantly longer length of critical care unit stay compared with all other causes, and the absolute risk of maternal mortality was the largest compared with all other causes, comprising 41% of women who died from severe sepsis. Despite the significant influenza epidemic, which occurred from 2009 to 2010, only 10.5% (27/257) of pneumonia/respiratory infection cases were identified as being due to the pandemic AH1N1 strain, although reporting is likely to be incomplete. Incidence of primary pneumonia (bacterial and viral) is known to increase during seasonal and epidemic influenza periods,^24^ which is reflected in the increased proportion of severe sepsis from respiratory infection from 2009 to 2010. However, in the pre-epidemic year of 2008, respiratory infection was also the largest source of severe maternal sepsis.

Between 2011 and 2012, it was found that the largest proportion of severe sepsis cases (31%) among women in UK maternity units was due to genital tract infection.^9^ The results of this study indicate that in addition to genital tract infection, respiratory infection is a major source of severe maternal sepsis irrespective of an influenza epidemic. This should be considered in the context that pregnant and peripartum women are at greater risk of developing respiratory infection^25^ and an increased incidence of invasive streptococcal infections (caused by *Streptococcus pneumoniae* and *Streptococcus pyogenes* (group A streptococcus)), occurring through community-acquired respiratory transmission, has also been recently recorded in the UK.^26^ Immediate implications are that in addition to precautions for genital tract sepsis, there is clearly a precedent to improve prevention and timely recognition of severe respiratory tract infection in pregnant and recently pregnant women.^4^ Most maternal deaths and critical illnesses from severe sepsis occur due to delay in recognition and diagnosis.^3^ Obstetric and front-line clinicians should therefore maintain a high index of suspicion, as well as alert pregnant and recently pregnant women to the possible severity of any infection, in particular the clinical symptoms of respiratory and genital tract infection and the dangers of delay in seeking medical care. The recent report from the Mothers and Babies: Reducing Risk through Audit and Confidential Enquiries in the UK (MBRRACE-UK) Confidential Enquiry into Maternal Deaths^4^ highlighted the following as symptoms and signs for women to be aware of: high temperature, chills and shivering, fast heartbeat, fast breathing, breathlessness, headache, severe abdominal pain and extreme sleepiness. From an economic perspective, an improvement in recognition before onset of severe infection would have a substantial effect on intensive care resource usage. The Confidential Enquiry also highlighted the importance of immunisation against influenza as a means of preventing respiratory infection in pregnant women,^4^ and this has been emphasised in public health campaigns.

In addition to the risk of severe maternal sepsis associated with respiratory infection, there was a clear gradient in the risk of severe sepsis associated with decreasing socioeconomic status. This represents a striking example of health inequities within a high-income country. Importantly, a significant proportion of women who had a caesarean section (a well-established risk factor for severe sepsis) were also of low socioeconomic status (64.3%). It is unlikely, however, that rates of caesarean section were simply higher among more deprived women in the population. In a cohort study of all women delivering in English hospitals, Alves *et al*^27^ found that the most affluent women had a significantly higher odds of caesarean section compared with women who were most deprived. Implications of our finding, therefore, are that poorer women in general in the UK, and potentially poorer women who have a caesarean section, are at increased risk of severe sepsis. It is important to note that the temporality of infection, whether the infection occurred after the caesarean section or if the woman had a caesarean section as a result of antepartum infectious morbidity, could not be determined from these data. Each scenario has significant causal implications. This is particularly relevant given that a social gradient in the risk of severe maternal sepsis appears to be consistent with an overall social gradient in health outcomes that exists in the UK and other advanced-economy countries.^28^ While the mechanism behind this finding is unknown, women in more deprived geographic areas are known to have higher rates of poor underlying health^28^ and decreased uptake and continuity of maternity care.^29^ There is a need for further work to investigate causative and potentially modifiable factors relating to maternal sepsis among the most deprived.

Known risk factors for maternal sepsis morbidity supported by this study are: younger^30^ and older age,^6^ multiple gestation birth^6^ and caesarean section.^6^ ^23^ ^30^ Additionally, we interpret the significantly higher rate of stillbirth in this cohort to be primarily an outcome of severe maternal infection. Some of these effects, such as the observed association with younger maternal age, may be mediated through other factors such as deprivation. However, although the younger women had a higher rate of admission with sepsis, it is important to note that they had the lowest morality rate. Significant unadjusted physiological risk factors for severe maternal sepsis mortality are indicative of the continuum of sepsis severity, eventually leading to multiple organ failure and death.^31^ The risk factor of age ≥35 years should be evaluated in an adjusted model for mortality.

## Conclusions

This study shows that the burden of severe sepsis on pregnant and peripartum women admitted to critical care is significant. The finding that pneumonia/respiratory infection caused the largest proportion of severe sepsis cases and maternal sepsis deaths, irrespective of epidemic influenza, indicates a critical need to improve timely recognition of severe respiratory tract infection, in addition to genital tract infection, in the obstetric population. Further studies should elucidate the respiratory causative organisms to which this population is particularly susceptible. The social determinants that potentially play a role in prepartum and postpartum maternal sepsis mortality must also be clarified using multivariable risk modelling in order to redress inequalities in maternal health. The clear social gradient associated with risk of severe sepsis morbidity and mortality raises important questions regarding maternal health service provision and usage, and may need to be addressed at the health policy level.
